# Washing load influences the microplastic release from polyester fabrics by affecting wettability and mechanical stress

**DOI:** 10.1038/s41598-021-98836-6

**Published:** 2021-09-30

**Authors:** Michela Volgare, Francesca De Falco, Roberto Avolio, Rachele Castaldo, Maria Emanuela Errico, Gennaro Gentile, Veronica Ambrogi, Mariacristina Cocca

**Affiliations:** 1 Institute for Polymers, Composites and Biomaterials, National Research Council of Italy, Via Campi Flegrei, 34, 80078 Pozzuoli, NA Italy; 2 Department of Chemical, Materials and Production Engineering, University of Naples Federico II, P.Le Tecchio, 80, 80125 Naples, Italy; 3 Present Address: School of Geography, Earth and Environmental Sciences, University of Plymouth, Plymouth, PL4 8AA Devon UK

**Keywords:** Environmental sciences, Materials science

## Abstract

Microplastics released from textiles during the washing process represent the most prevalent type of microparticles found in different environmental compartments and ecosystems around the world. Release of microfibres during the washing process of synthetic textiles is due to the mechanical and chemical stresses that clothes undergo in washing machines. Several washing process parameters, conditions, formulations of laundering additives have been correlated to microfibre release and some of them have been identified to affect microfibre release during washing process, while no correlation has been evaluated between microfibre release and washing load. In the present study, microfibre release was evaluated as function of the washing load in a real washing process, indicating a progressive decrease of microfibre release with increasing washing load. The quantity of released microfibres increased by around 5 times by decreasing the washing load due to a synergistic effect between water-volume to fabric ratio and mechanical stress during washing. Moreover, the higher mechanical stress to which the fabric is subjected in the case of a low washing load, hinders the discrimination of the effect on the release of other washing parameters like the type of detergent and laundry additives used.

## Introduction

Environmental pollution due to microplastics, plastic fragments with dimensions lower than 5 mm, represents a global problem that has become particularly relevant in recent years^1^. In 2017, it was estimated that microplastics released from textiles during the washing process, named “microfibres”, contributes by about 35% to the global ocean pollution of primary microplastics^2^. For the first time in 2011^3^, the presence of synthetic microfibres in the environment was correlated to the laundering of synthetic clothes. Release of microfibres during the washing process of synthetic textiles is due to the mechanical and chemical stresses that clothes undergo in washing machines, and due to their dimensions, not all microfibres released can be blocked by wastewater treatments plants (WWTPs)^4^, reaching in this way seas and oceans and constituting a risk for the ecosystem. Several studies investigated the release of microfibres during the washing process of synthetic textiles, using different procedures. Using a multistep filtration procedure of the whole volume of water involved during a real washing test and the gravimetric determination of the amount of released microplastics, it was estimated that the release ranges from 124 to 308 mg of microfibres per kg of washed fabric depending on the textile structure of washed garments^4^, corresponding to a number of microfibres ranging from 640,000 to 1,500,000. Moreover, using a filtration procedure of the whole volume of washing water with a 25 μm pore size filter directly attached to the end of the drain hose, it was estimated that a washing load of about 6 kg of acrylic fabrics releases more than 700,000 microfibres in a single washing process^5^.

Synthetic microfibres were identified in sediment samples from shorelines^3^, seas^6^ and riverbank^7^, terrestrial and freshwater ecosystems^8^, indoor environments^9^, food^10^, drinking water^11^, as well as in the digestive tracts of many aquatic^12–15^ and terrestrial organisms^16^. In light of the fact that microfibres are abundantly present in the environment, evaluations of their toxicity for fish and deep-sea organisms were conducted in several studies. The effect of microfibres ingested by the crab *Carcinus maenas,* was investigated in 2015^17^, and it was found a decrease in the growth process in term of reduction of energy available. In this study, crabs ingested plastic microfibres added in different concentration to their food (from 0.6 to 2.0 mg added to 2 g of the feed) and showed a reduced scope for growth, mainly correlated to a reduction in food consumption. A study conducted in 2016^18^ on the exposure of *Daphnia magna* to microfibres (from 12.5 to 100 mg/L), showed an increase of the mortality of the species. The interaction of microfibres with digestive tracts of *Gammarus duebeni* was recently reported^19^. Despite no apparent short-term effects on amphipods, the fluorescence microscopy analysis of digestive tracts showed that half of the species that had been exposed to microfibres had accumulated fragments in the internal systems. These amphipods are key species as aquatic food, so the possible accumulation of anthropogenic microfibres in their digestive system has potentially further implications.

In such scenario, it is important to evaluate the quantity of microplastics that can be released from synthetic clothes during the laundering process and to correlate the microplastic release to detergent formulation/washing process/textile structure, in order to identify relationships and possible parameters of influence. Several works investigated the role of additives (detergent, softener)^5,20–25^, washing temperature and washing time^5,21–23,25–29^, different textiles parameters^22,23,30,31^ and the effects of water-volume on microfibres release^29^. In particular, many studies investigated the role of different compositions of fabric: the most investigated textile was polyester, the dominant synthetic fibre on the textile market^32,33^, both in laboratory simulations of washing tests^21–23,29–31,34^ and in household washing machines^4,5,20,21,24–29,35–38^, but also recycled polyester and virgin polyester^39,40^, polyamide^20,28,34,35,38^, acrylic^5,20,30,34,35,38^, cotton fabrics^20,21,25,26,35,37^, blends of polyester/cotton^4,5,21,26,41^ and blends of polyester/elastane^36^. The general trend confirmed that the detergent caused a higher release with respect to washing tests performed with only water^21–23,28,34,42^ and the greatest release was estimated in the case of natural or artificial fibres compared to synthetic ones^4,9,20,21^. Another trend verified in several studies was the decreasing release of microfibre from textiles in subsequent washes^4,5,20,21,24,36,38^, which could be explained considering that in the first washes garments shed more microfibres due to the presence of leftovers from the garment manufacturing process^36^.

Moreover, several studies investigated the release of microfibres from different types of garments using different washing loads in a real washing machine, highlighting that the washing load could significantly influence microfibres release. In some cases, real washing loads were tested (e.g. De Falco et al.^4^: a constant load of about 2–2.5 kg; Zambrano et al.^21^: a load of about 1.8 kg; Lant et al.^25^: a variable load that ranged from 1.0 to 3.5 kg and 3.5–6.0 kg; Dalla Fontana et al.^27^: a constant load of about 2 kg; Kelly et al.^29^: 1.5 ± 0.01 kg; Galvão et al.^35^: a load that ranged from 1.83 to 5.57 kg) and in other studies washing tests were carried out with pieces of fabric (e.g. Napper et al.^5^:20 × 20 cm; Yang et al.^28^: rectangular pieces of 1.5 m × 0.6 m). Others works carried out washing tests on a single garment/blanket^20,24,36,43^. The differences in the methods applied to quantify microfibre release and especially in parameters like washing load, make difficult the comparison between the results and may compromise the evaluation of the influence of a particular parameter on the release.

The present work aims to identify the relationship between microfibre release and washing load and to clarify the role of the washing load on the release, through demonstrating two main hypotheses: (1) washing load has an effect on microfibre release, (2) the washing load hides/amplifies the effect of other parameters on the release. For these purposes, washing trials with four different loads in the same washing conditions were performed. The influence of the washing load on other parameters was demonstrated by carrying out trials with different detergents. For each washing trial, the whole volume of wastewater was filtered through subsequent filters with decreasing pore size, allowing the determination of the mass and dimensions of microfibres released.

## Results

### Effect of the washing load

**Figure 1:**
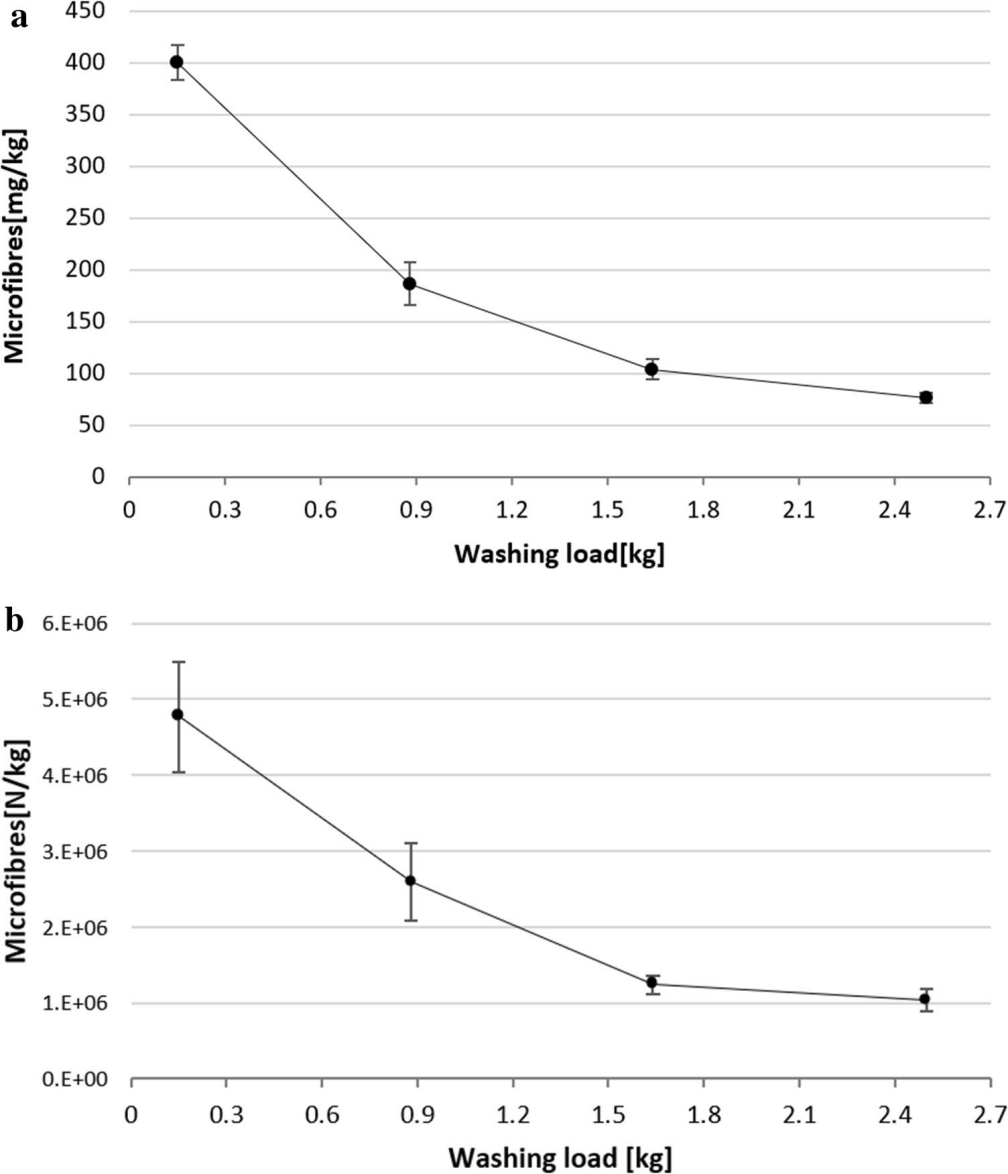
(**a**) Microfibres released per kg of washed fabric (expressed in mean mg/kg ± SD, n = 2) during washing process performed with 0.15 kg, 0.88 kg, 1.64 kg and 2.50 kg. (**b**) Number of microfibres released per kg of washed fabric (expressed in mean N/kg ± SD, n = 2) during washing processes performed with 0.15 kg, 0.88 kg, 1.64 kg and 2.50 kg.

Comparing the results obtained during washing trials performed in a real washing machine with different washing loads, it was possible to evaluate the effect of the release as a function of the load. The amount of microfibres released per kg of washed fabric (mg/kg) during the washing test was calculated to range from 401 ± 17 to 76 ± 5 mg/kg, depending on the washing load used as reported in Fig. 1a. The results indicate that 401 ± 17 mg/ kg of microfibres were released in the washing tests performed using a washing load of about 0.15 kg (N. 1 T-shirt), 187 ± 21 mg/kg in the tests performed with a washing load of about 0.88 kg (N. 6 T-shirts), 104 ± 10 mg/kg in the tests with a washing load of about 1.64 kg (N. 11 T-shirts) and finally 76 ± 5 mg/kg in the tests with a washing load of 2.50 kg (N. 17 T-shirts).

**Figure 2:**
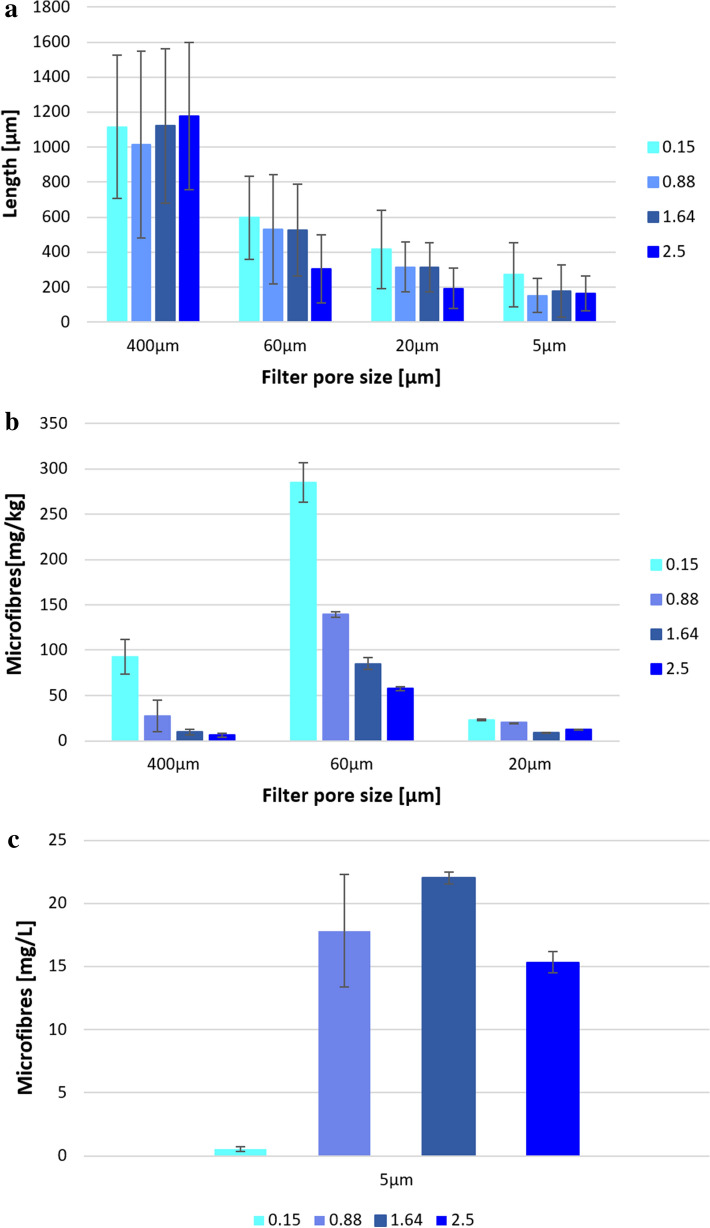
(**a**) Lengths of microfibres recovered on 400 μm, 60 μm,20 μm and 5 μm pore size filters (n = 30); (**b**) amount of microfibres recovered on 400 μm, 60 μm and 20 μm pore size filters (expressed in mg/kg ± SD, n = 2); (**c**) microfibres recovered on 5 μm pore size filters (expressed in mg/L ± SD, n = 2).

The number of microfibres released, calculated from the amount of microfibres using the formula reported in the methods section, is reported in Fig. 1b. Microfibres released during the performed washings tests were found to decrease from 4,766,338 N/kg, using the lowest washing load, to 1,041,736 N/kg, value obtained using a load of 2.50 kg. From the results it was evident that the washing load strongly affects the extent of microfibre released from garments during washing processes. These results were obtained with a limited number of replicates (n = 2) inhibiting statistical analysis. The different washing loads tested in the washing trials implied the usage of a great amount of clothes per test, that ranged from 1 to 17 T-shirts per each replicate and the usage of a total of 80 t-shirts, complicating the number of replications affordable. It is necessary to highlight that the use of such a low number of replicates (n = 2) represents a limitation to statistically validate the obtained results. Nevertheless, the high differences in the data and the low standard deviations allow to identify a clear trend in the release and to highlight that a lower washing load negatively affects microfibers shedding. A similar approach in number of replicates and related standard deviation was already applied in a previous work^4^. The usage of a multistep filtration procedure allows to recover microfibres according to their dimensions on the used filters having different pore sizes. The analysis of the optical micrographs of the microfibres recovered on the filters, reported in Figure S1 of the Supporting Information (SI), allowed to determine that microfibres with an average length ranging from 1013 to 1177 μm were blocked on the 400 μm pore size mesh, those recovered on 60 and 20 μm pore size filters presented an average length of 306–597 μm and of 193–416 μm, respectively; finally, microfibres with length range of 153–272 μm were recovered on 5 μm pore size filters (Fig. 2a). Statistical analysis pointed out significant difference among the lengths of microfibres released during washings using all the loads (KW 2.50–0.88 kg: *p* = 0.013; KW 2.50–1.64 kg: *p* = 0.001; KW 2.50–0.15 kg: *p* = 0.000; KW 0.88–0.15 kg: *p* = 0.000; KW 1.64–0.15 kg: *p* = 0.000; Tables S1, S2) except among microfibres deriving from intermediate loads, 0.88 and 1.64 kg (KW 0.88–1.64 kg: *p* = 0.367; Tables S1–S2). Microfibre diameters were calculated to be 11.5 ± 2.2 μm for the washing load of 0.15 kg, 11.8 ± 2.4 μm for 0.88 kg, 12.3 ± 2.7 μm for 1.64 kg and 12.3 ± 2.5 for 2.50 kg*.*

In general, the greatest amount of microfibres was recovered on 60 μm pore size filters, approximately 75% of the total quantity released during the wash (Fig. 2b). The amount of wastewater filtered through 5 μm pore size filters was recorded to have an indication of the amount of microfibres retained on the smallest pore size filter (Fig. 2c).

### Influence of washing load on the effect of detergent type

**Figure 3:**
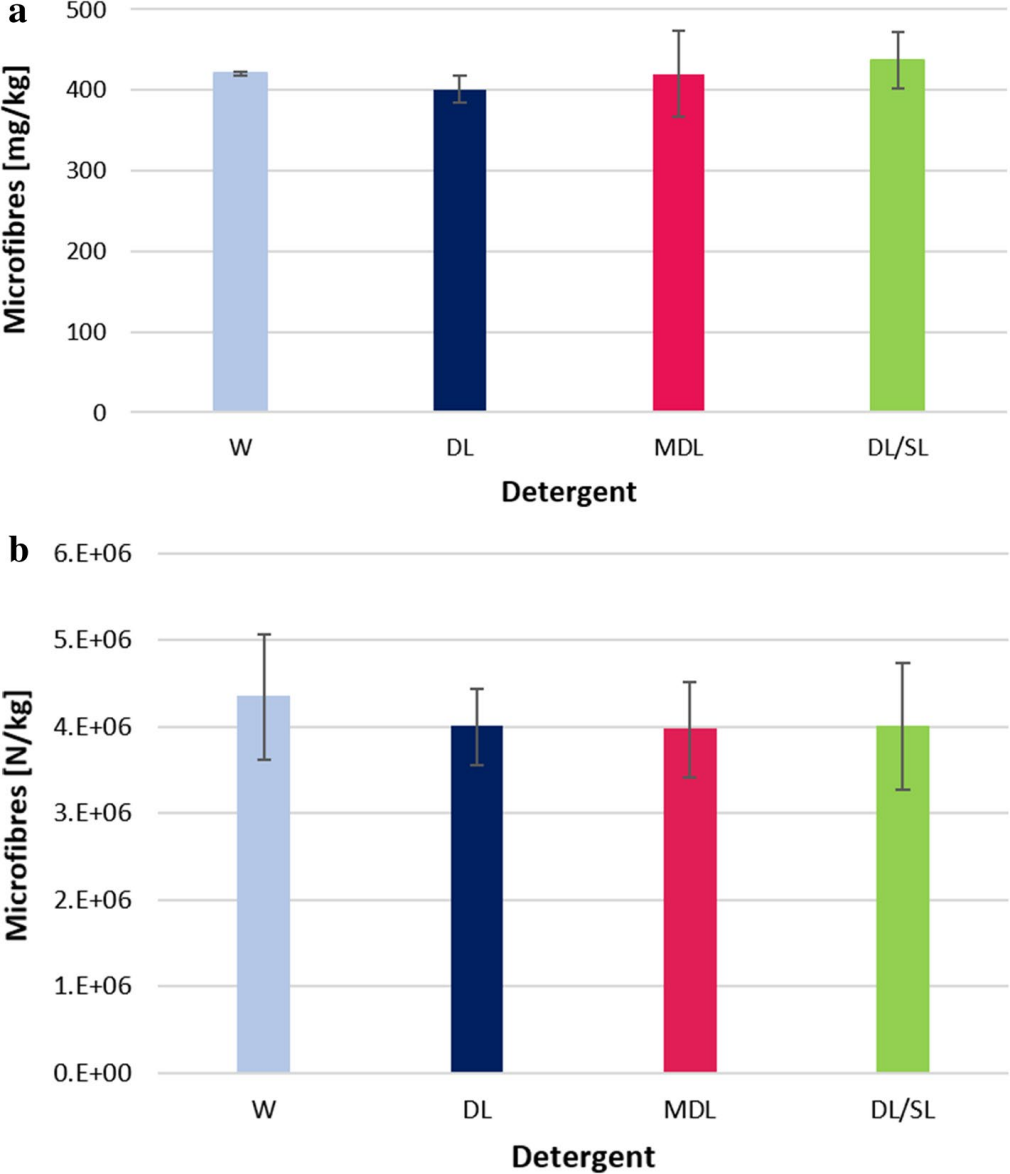
(**a**) Microfibres released per kg of washed fabric (expressed in mean mg/kg ± SD, n = 3) during washing processes performed with water (W), commercial liquid detergent (DL), mild liquid detergent (MDL) and combination of the commercial liquid detergent and commercial softener (DL/SL). (**b**) Number of microfibres released per kg of washed fabric (expressed in mean N/kg ± SD, n = 3) during washing processes performed with W, DL, MDL and DL/SL.

In order to demonstrate how washing loads amplify or hide the effect of the detergent on microfibre release during washings, different combinations of laundry additives were used and tested in washing trials using the lowest washing load. The release of microfibre per kg of washed T-shirt during washing test performed with only water was calculated to be 420 ± 2 mg/kg , 401 ± 17 mg/kg for washings performed using a commercial liquid detergent (DL), 420 ± 54 mg/kg in washing trials performed with mild detergent (MDL); 437 ± 35 mg/kg when a combination of commercial liquid detergent and commercial liquid softener (DL/SL) was used, as reported in Fig. 3a. No significant difference was detected among the quantities of microfibres released per kg of washed fabric for each type of detergent (*F*(3,3) = 1.085, *p* = 0.464; see SI–Table S3).

**Figure 4:**
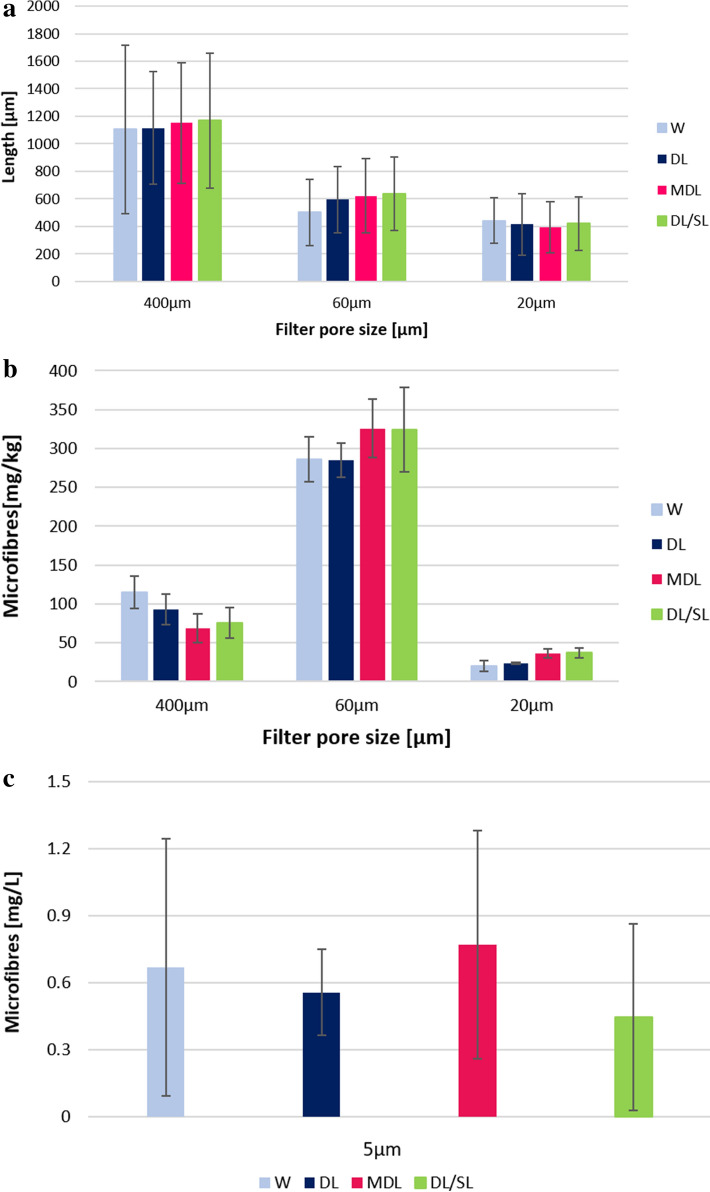
(**a**) Lengths of microfibres recovered on 400 μm, 60 μm,20 μm and 5 μm pore size filters (n = 30); (**b**) amount of microfibres (mg/kg) recovered on 400 μm, 60 μm and 20 μm pore size filters (n = 3); (**c**) microfibres recovered on 5 μm pore size filters (expressed in mg/L ± SD, n = 3).

Also in this case, the quantities determined were converted in number of microfibres released during the washing tests: the number of microfibres per kg of washed fabric were 4,341,793 for washing trial performed using only water, 3,997,912 N/kg when DL was used, 3,963,859 N/kg and 4,005,603 N/kg for MDL and DL/SL, respectively (Fig. 3b). No significant differences were detected among the data of the tests (*p* = 0.933—KW). Microfibres with a length that ranged from 1106 to 1170 μm were blocked by the 400 μm pore size mesh; the 60 μm pore size filter blocked fibres with a length ranging from 501 to 638 μm; fibres with a length ranging from 396 to 441 μm were blocked by 20 μm pore size filter (Fig. 4a). The greatest amount of microfibres was recovered on the filter of 60 μm pore size, approximately 72%, as reported in Fig. 4b. The amount of microfibres retained by the smallest pore size filter (i.e. 5 μm) is reported in Fig. 4c. No significant difference was detected among the lengths of the microfibres released by using the different detergent types (*p* = 0.240—KW). The average length of microfibres recovered from the washing trial performed with only water was evaluated to be 683 ± 493 μm, 706 ± 423 μm for DL, 721 ± 446 μm and 733 ± 464 μm for MDL and DL/SL, respectively. The average diameter of the microfibres recovered from the filters was 11.7 ± 1.9 μm for microfibres released during the washing tests performed with W, 11.5 ± 2.1 μm by using DL, 11.7 ± 2.1 μm for microfibres released with MDL and 11.7 ± 2.3 μm for those released from washing tests performed by using DL/SL.

## Discussion

The results of the washing tests performed using different washing loads indicate that the load strongly affects the release. In detail, the amount of microfibres released per kg of washing load decreased by increasing the washing load. Such results could be due to a synergistic effect between the water-volume to fabric ratio and mechanical stress on the fabric, at which the different amounts of fabrics are exposed during washing. This synergistic effect was responsible of the considerably high quantity of microfibres released from the washing of a single garment, despite previous works have pointed out that yarns made of continuous filaments tend to release less microfibres than those composed by short staple fibers^4,9,22,27,34^. The laundry process of the tested knitted fabric, made of yarns arranged in a loose structure with low twist, resulted in a relaxation state that led to a more open structure of the garment, eventually favouring microfibre release. In fact, washing tests carried out with a single garment led to a greater wettability of the fabric that could enhance the mobility of microfibres that detach from the yarn. The greater wettability is also ascribable to the large water-volume to fabric ratio used in the washing processes of a single T-shirt, e.g. 40 l for a load of 0.15 kg, which is higher than the 50 l used for the 2.5 kg load. This consideration is consistent with other works^9,29^, which pointed out that a higher water-volume to fabric ratio is responsible for a greater release of microfibres. In another work^25^, similar results were obtained; in particular, a release of 132 mg of microfibres per kg of washed fabric was recorded in the tests performed using a mass range of 1.0–3.5 kg. This amount was higher than 66 mg of microfibres per kg of washed fabric obtained with a mass range of 3.5–6.0 kg. However, the detergent used in their washing tests was a pod detergent that could led to different considerations. Different washing loads of 100% polyester garments, from 0.180 to 0.74 kg, were tested by Kärkkäinen et al.^38^ using a household washing machine representing loads comparable with those tested in the present work (particularly 0.150 kg and 0.88 kg). They found a microfibre release ranging from 3.1 × 10^6^ kg^−1^ to 1.8 × 10^5^ kg^−1^, that are in agreement with the release here reported (4,766,338 N/kg for the washing load of 0.150 kg and 2,593,031 N/kg for the washing load of 0.88 kg). Unfortunately, the cited work did not point out differences among the values of microfibres released per kilogram of washed fabric as a function of washing load, since it was aimed to evaluate the effect of others washing parameters, such as type of material, type of fabric and tumble drying. Belzagui et al.^36^ carried out washing trials in order to evaluate the release from different fabrics and obtained the highest microfibre release of 560MF/g from an acrylic-polyamide garment using a wash load of 74 g, followed by polyester-elastane and other polyesters fabrics (175 MF/g using mass range of 101–728 g). Galvão et al.^35^ obtained a microfibre release between 1842 and 6259 MF/g testing washing loads ranging from 1.83 to 5.57 kg of garments with different composition (cotton, polyester, polyamide, viscose, elastane and acrylic). Contrary to the trend obtained in the present paper, they observed a higher microfibre release in washing tests performed using a greater number of synthetic fabrics. In both cases, the reported microfibre release was not directly correlated to the washing load, but to other washing parameters such as different garments composition (e.g. cotton, polyester, polyamide, elastane), subsequent washes and type of fabric (woven and knitted). Fabric structure strongly influences microfibres detachment during the laundry processes^4^. To reduce microfibre release, compact structures, such as woven, using highly twisted yarns made of continuous filaments could be preferable to more loose structure, including knitted, with yarns made of short staple fibres and low twist^9^. In Table 1, a comparison of literature data with the results obtained in the present manuscript is reported.

**Table 1:** Comparison of the results of the present work with previous studies on microfibre release as a function of washing load.

Study	Type of fabric	Microfibre released	Washing load
Lant et al.^25^	100% polyester garments	132 mg/kg 66 mg/kg	1.0–3.5 kg 3.5–6.0 kg
Kärkkäinen et al.^38^	100% polyester garments	3.1 × 10^6^ kg^−1^–1.8 × 10^5^ kg^−1^	0.18–0.74 kg
Belzagui et al.^36^	Acrylic-polyamide, polyester-elastane, polyester garments	560 MF/g 175 MF/g	74 g 101–728 g
Galvão et al.^35^	Cotton, polyester, polyamide, viscose, elastane, acrylic	1842–6259 MF/g	1.83–5.57 kg
Present work	100% polyester T-shirts	401–76 mg/kg (4.8 × 10^6^ kg^−1^–1.0 × 10^6^ kg^−1^)	0.15–2.50 kg

As mentioned in the introduction, there are several washing parameters that should be taken into consideration affecting microfibre release, such as the increase in washing temperature, washing time^5,21–23,26–29^ and mechanical action^27,29^, which enhance the mechanical stresses that garments undergone during the washes and, consequently, increase the mass of microfibres released during the washing cycle^21,26–28^. It is known that the movement of the fabrics inside the drum of the washing machine yields a large amount of mechanical stress onto fabric, such as flow through the yarn that induces pressure drops across the fabrics and enhances shear forces due to fabrics rubbing together^45^. There are many other parameters that could be taken into consideration to investigate how to reduce microfibre shedding. In this respect, the present study represents the first effort carried out to directly correlate the microfibre release as a function of the washing load in a real washing process, and to quantify the benefits of a full washing load, in term of the reduction of microplastic shedding, and energy consumption^46^.

In addition, the extent of how the washing load could invalidate the effect of laundry additives on microfibre release was also evaluated, using a single T-shirt and different liquid detergents (commercial detergent, mild detergent, and commercial detergent in combination with a softener). The results suggest that the role of the type of detergent used, as well as that of the softener on the release of microfibres from the T-shirt, cannot be detected since the high release due to the usage of a lower washing load hinders the determination of other effects on the release. The hypothesis that the mechanical stress strongly increases microfibre release was evaluated in other works^21,22^ that pointed out the negative effect of the mechanical action during the washing process, using small-scale tests. Moreover, high moisture levels, which are of course present during the washing process using a low washing load, strongly influence the mechanical behaviour of synthetic and naturals fibres^47^. The highest mechanical stress that the garment underwent in the case of a low washing load (of about 0.150 kg) did not allow to differentiate the effect of the laundry additives. In the washing tests carried out with a washing load of a single garment, the obtained amounts of microfibres released were absolutely comparable, both with and without detergents. Washing tests were performed also with the combination of commercial liquid detergent and a commercial softener, confirming this trend. These results are strongly affected by the low washing load since in several works is reported that detergent promoted the release of microfibres from the fabrics, both in a household washing machine^28^, and in a laboratory simulator^21–23,34,42^. Nevertheless, the trend obtained was in line with other works^24,25^ that concluded that additives in the washing process did not significantly influence the emission of microfibres. However, such studies present some differences with the present work. In particular, Lant et al.^25^ evaluated the impact of the detergent and of the fabric softener by washing three or four fleeces or 10 T-shirts in a real household washing machine, whereas Pirc et al.^24^, tested a washing load comparable to the present work, of about 320 g, in a domestic washing machine, confirming that with a low washing load, additives were not a main factor in fibres release, but rather a mechanical stress. They also investigated the role of the combination of the detergent and a softener, assessing that with the used load these additives in washing process did not significantly influence the emission in microfibres, in agreement with the results here reported. A further correlation was found in another work^5^ published in 2016, that evaluated the release of microfibres from washing processes in a domestic washing machine of 20 cm × 20 cm squares cut by different fabric types (polyester, polyester-cotton blend, acrylic) using different combinations of detergents (no detergent, bio-detergent, non-bio detergent, conditioner). The authors did not observe a consistent pattern for the effect of the detergent, probably due to the low washing load used in the tests, since, even in this case, the washing load tested was lower than a real load in a laundry process. Recently, an unconventional pattern was obtained by Cesa et al.^20^, who tested low washing loads (composed by a single garment) in presence and absence of detergent. They found a reduction of the microfibre release when detergent was used. This tendency was ascribed to the presence of foam, generated by surfactants, that can reduce washing mechanical action and induce foam adsorbing by fibres surface, mitigating fibre damage^22,23^. On the other hand, detergents decrease the surface tension, improving the wetting of the fabric and facilitating particle liberation^34^. In this sense, detergents may behave differently and lead to different results, according to the methodology used.

Regarding the dimensions of microfibres released from the washing trials performed with different washing loads, the length ranged from 26 to 3029 μm and most of the released fibres had a size between 200 and 600 μm, with few microfibres with a dimension lower than 100 μm and the longest fibre of about 3 mm. The observed lengths were evaluated to be comparable with dimensions reported by Zambrano et al. ^21^ with an average length > 200 μm and few microfibres of about 2.5 mm, and slightly lower than Kelly et al.^29^, that calculated an average length of 0.96 mm, with the longest fibre recorded at 4.91 mm. The total average length was calculated to be 549 ± 451 μm consistent with the value reported in another study which carried out the filtration procedure through a single filter with different pore sizes (Yang et al.^28^: with a 5 μm filter pore size found an average length of polyester fibres of 499 ± 506 μm). Although the average length was 10 times lower than average length calculated by Napper et al.^5^ and Pirc et al.^24^, the larger fibres were longer than the general values found in many works^23,31,35–37,42^, generally based on observation of a limited number of fibres.

The average length of microfibres shed during washing with a load of 0.15 kg, (628 ± 425 μm) was greater than the average length calculated for the washing process performed with higher loads (499 ± 452 μm with the load of 0.88 kg; 534 ± 454 μm with the load of 1.64 kg; 459 ± 480 μm with the load of 2.50 kg). This trend is in conflict with the considerations of another work^21^, for which higher mechanical action break off small microfibres.

This work demonstrate that the washing load strongly affects microfibre release during washing of polyester T-shirts. The observed trend, i.e. microfibres release increases with decreasing washing load, allows to identify the main role on the release of the mechanical action during the washing process in combination with high moisture levels, generally present during washing using a low washing load. Low washing load hides the effect of the usage of different detergents and different laundry additives on the release since using a low washing load, detergents and additives were not a main factor in microfibres release. The influence of the washing load should be considered to estimate the extent of microfibres pollution in the environment and to account for the high variability of the data reported in literature. Moreover, the effect of the washing load should be taken into account to develop adequate mitigation approaches to protect the fabric from the chemical and mechanical actions of laundering^48–50^.

## Methods

### Materials

The garment selected for the washing tests was a commercial blue T-shirt made of 100% polyester, kindly supplied in more items by Plastic Soup Foundation (Amsterdam, the Netherlands). In order to evaluate the textiles parameters of the T-shirts, an optical microscopy analysis with a LEICA M205C light microscope was carried out. The T-shirt was made of knitted polyester characterized by a low hairiness, which consists of small fibres that protrude from the yarn, and the yarns were constituted of continuous filaments with low twist. The liquid detergent used for the washing test was a commercial detergent widely used in domestic laundry. In order to evaluate the influence of the washing load on the effect of detergent, washing tests were also performed using a commercial liquid detergent (DL), a mild detergent (MDL) and DL in combination with a commercial softener (SL). The detailed compositions of the detergents used are reported in the SI. The detergents were used in the dose recommended by the supplier.

### Washing/filtration protocol and gravimetric method

The washing tests were performed using a front-loading Bosch washing machine Serie 4 Varioperfect WLG24225it. The selected washing program was for synthetic clothes using 40 °C, 1200 rpm and a washing time of 1 h 47 min. The washing machine automatically regulated the amount of water used during the washing according to the washing load. The water volume used in the washes was measured as function of the washing load and it ranged from 40 l for the load of 1 T-shirt to 50 l for 17 T-shirts. Each washing test was performed on pre – washed garments. The prewashing was performed with the same washing program selected for the washing trials. In order to evaluate the effect of the washing load, washing tests were conducted on different numbers of the same type of T-shirts. In particular, four different washing loads were tested, each test was performed in two replicates:

- N. 1 T-shirt that corresponded to a washing load of about 0.15 kg
- N. 6 T-shirts corresponding to a washing load of about 0.88 kg
- N. 11 T-shirts that corresponded to a washing load of about 1.64 kg
- N. 17 T-shirts corresponding to a washing load of about 2.50 kg

Additional tests were performed in triplicates with a washing load of 1 T-shirt, using the three detergent combinations selected (DL, MDL and DL/SL) and washing also with only water (W) as reference.

For each washing test, the whole volume of wastewater of the washing machine underwent a multistep filtration procedure previously developed^51^. Firstly, the wastewater coming directly from the drainpipe was filtered through a 400 μm pore size mesh with a diameter of 6 cm and recovered in tanks. Then, the wastewater was further filtered by means a peristaltic pump (SP 311/60 Velp Scientifica) connected with Tygon tubes with subsequent filtrations through three different pore size filters: 60 μm pore size nylon filter of a diameter of 4.7 cm (Merck Millipore), 20 μm pore size nylon filter with a diameter of 4.7 cm (Merck Millipore), and 5 μm pore size nylon filter with a diameter of 4.7 cm (Durapore ®, Merck Millipore). To avoid clogging problems, for the last filter with pore size of 5 μm, only an aliquot of about 300 ml of wastewater was filtered. At the end of each tack, 1L of distilled water was poured into, the tank was shaken, and the water filtered. This procedure was carried out twice for each tank to remove possible fibres that remained attached on the surface of the tanks. After each filtration, about 1L of distilled water at 70 °C was fluxed through the filter to remove the excess of detergent. All the filters were dried at 105 °C for 1 h and weighted before and after filtration to evaluate the amount in grams of microfibres per kg of garment washed (M_t_). Subsequently, the number N of microfibres released to the wastewater from each washing test was estimated using the equation reported below, assuming that all fibres were of cylindrical shape ^4,5^

$$\mathrm{N }=\frac{\frac{{\mathrm{M}}_{\mathrm{t}}}{\uprho }}{\uppi \cdot \frac{{\mathrm{D}}^{2}}{4}\cdot \mathrm{L}}$$

where M_t_ is the weight of microfibres released during the washing test, ρ is the density of the material, L is the mean microfibre length and D is the mean microfibre diameter. Length and diameter of the fibres were determined by using a LEICA M205C light microscope and analysing the acquired micrographs by ImageJ (release 1.43u). A mean value was calculated for length and diameter based on the observation of about 120 fibres per sample.

### Quality assurance and quality control (QA/QC)

Cotton lab coats and nitrile gloves were worn during all the experimental work. Cross-contamination of microfibres between washes was prevented by running, after the end of each washing test, an empty washing cycle at 40 °C, 400 rpm and 30 min. To avoid cross contamination of microfibres among the different filtrations, Tygon tubes, filter holders and tanks were cleaned with distilled water and with a jet of compressed air. Blank tests were performed in triplicates by running empty washing cycles in the same washing conditions, i.e. program for synthetics at 40 °C, 1200 rpm and 1 h 47 min. The wastewater obtained from these blank tests was filtered using the same procedure described above. The weight of the filters obtained from the washing tests were blank corrected by subtracting the average weight of the filters obtained from the blank tests. Furthermore, three procedural blanks were run during sample processing to monitor possible contamination. During the filtration procedure, the filter was exposed to possible air contamination only during the operations of weighing, insertion in the filter holder, removal from the filter holder and storing in a petri dish. Therefore, filter papers with a diameter of 4.7 cm (Whatman™) were placed into petri dishes in the laboratory near the balance board and on the table were the filtration occurred. The petri dishes were opened during the operations mentioned above and then, the filter papers were observed using a LEICA M205C light microscope to check for the presence of contaminants. The results showed the presence of cellulosic fibres in very low quantity (n. 1 fibre in petri dish on the table, and n. 2 fibres in the petri dish positioned near the balance). The contamination was considered negligible.

### Statistics

Statistical analysis of the amount, length and number of microfibres released during washing processes was carried out by using IBM® SPSS® Statistics software. The data were tested for normality using a Shapiro–Wilk test and for homogeneity of variance by using Levene's test. One-way Analysis of Variance (ANOVA) was performed to assess any significant differences among the data acquired. Kruskal–Wallis (KW) test was performed when data did not comply with the assumption of normality; Welch ANOVA test was performed when data did not comply with the assumption of homogeneity of variances. A 5% significance level was used for all statistical tests; *p* values < 0.05 indicate significant difference among the data.

## Supplementary Information

Supplementary Information.
