# Supplementary material for: Washing load influences the microplastic release from polyester fabrics by affecting wettability and mechanical stress
Source: Sci Rep. 2021 Sep 30;11:19479. doi: 10.1038/s41598-021-98836-6 (PMC8484352; doi:10.1038/s41598-021-98836-6)
Supplement: Supplementary file 1 — Supplementary Information. [file 41598_2021_98836_MOESM1_ESM.pdf]

Supporting information

Washing load influences microplastic release from polyester fabrics by affecting wettability and mechanical stress

Michela Volgare[1,2], Francesca De Falco[1*], Roberto Avolio[1], Rachele Castaldo[1], Maria Emanuela Errico[1], Gennaro Gentile[1], Veronica Ambrogi[2], Mariacristina Cocca[1*]

1 Institute for Polymers, Composites and Biomaterials – National Research Council of Italy Via Campi Flegrei, 34 - 80078 Pozzuoli (NA) Italy.

2 University of Naples Federico II, Department of Chemical, Materials and Production Engineering, P.le Tecchio, 80 – 80125 Naples, Italy

* corresponding authors:

Francesca De Falco, Institute for Polymers, Composites and Biomaterials – National Research Council of Italy Via Campi Flegrei, 34- 80078 Pozzuoli (NA) Italy, e-mail francesca.defalco@plymouth.ac.uk

Mariacristina Cocca, Institute for Polymers, Composites and Biomaterials – National Research Council of Italy Via Campi Flegrei, 34- 80078 Pozzuoli (NA) Italy, e-mail mariacristina.cocca@ipcb.cnr.it

Compositions of the detergents used in the washing tests.

- **Commercial liquid detergent (DL):** 5-15% anionic surfactants and non-ionic surfactants, <5% soap, phosphonates, optical brightener, benzisothiazolinone, methylisothiazolinone, enzymes and perfume (butylphenyl methylpropional, limonene).
- **Mild detergent (MDL):** water, sodium laureth sulfate, Glycereth-6 cocoate, sodium chloride, sodium formate, sodium lauryl sulfate, potassium cocoate, phenoxyethanol, Caprylyl/Capryl Wheat Bran/Straw Glycoside, tetrasodium glutamate diacetate, parfum, sodium carboxymethyl inulin, steardimonium hydroxypropyl hydrolysed wheat protein, polydimethylsiloxane.
- **Softener (SL):** water, distearoyltehyl/dipalmitoylethyl dimonium chloride, parfum, isopropyl alcohol, cocos nucifera oil, acrylic polymer, linalool, formic acid, hexyl cinnamal, eugenol, dipropylene glycol, monosodium etidronate, benzisothiazolinone, colorant, dimethicone, sodium hydroxide, trimethysiloxusilicate/dimethicone crosspolymer, glyceryl stearate, PEG-20 stearate, disodium phosphite, sodium hydrogen phosphonate, phosphorus acid, sodium salt, hydroxyethycellulose.

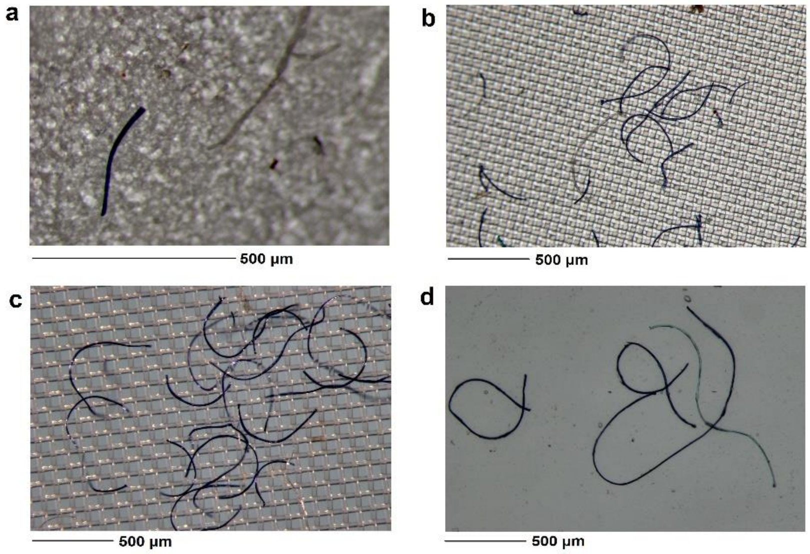

Figure S1. Optical micrographs at different magnifications of fibres recovered from: (**a**) filter with pore size of 5 µm, (**b**) filter with pore size of 20 µm, (**c**) filter with pore size of 60 µm, (**d**) filter with pore size of 400 µm

Table S1. Kruskal-Wallis test on lengths of microfibres recovered from filters after the washing processes performed with the washing load of a single T-shirt (0.15 kg), 0.88kg, 1.64 kg and 2.50 kg and b) its pairwise comparisons.

	Null Hypothesis	Test	Sig.	Decision
1	The distribution of L is the same across categories of Sample.	Independent-Samples Kruskal-Wallis Test	,000	Reject the null hypothesis.

Asymptotic significances are displayed. The significance level is ,05.

Table S2. Pairwise comparisons of the Kruskal-Wallis test on lengths of microfibres recovered from filters after the washing processes performed with the washing load of a single T-shirt (0.15 kg), 0.88kg, 1.64 kg and 2.50 kg and b) its pairwise comparisons.

Pairwise Comparisons of Sample

Sample 1-Sample 2	Test Statistic	Std. Error	Std. Test Statistic	Sig.	Adj. Sig.
2.50-0.88	73,026	29,333	2,490	,013	,077
2.50-1.64	99,428	29,333	3,390	,001	,004
2.50-0.15	206,665	27,327	7,563	,000	,000
0.88-1.64	-26,402	29,251	-,903	,367	1,000
0.88-0.15	133,639	27,240	4,906	,000	,000
1.64-0.15	107,237	27,240	3,937	,000	,000

Table S3. Welch - ANOVA on mg of microfibres released per kg of washed fabric during washing process performed with water (W), commercial detergent (DL), mild detergent (MDL) and commercial detergent in combination with softener (DL/SL)

mg/kg

	Statistics[a]	gl1	gl2	Sig.
Welch	1,085	3	3,386	,464

a. Asymptotically F distributed.
